# ﻿Four new species and four newly recorded species of *Omphale* Haliday (Hymenoptera, Eulophidae) from China, with a key to Chinese species

**DOI:** 10.3897/zookeys.1215.130669

**Published:** 2024-10-15

**Authors:** Ming-Rui Li, Jia-Sheng Wang, Ze-Ji Jing, Qing-Fan Meng, Hong-Rui Zhao, Xing-Peng Li, Sheng-Dong Liu, Cheng-De Li

**Affiliations:** 1 Jilin Provincial Key Laboratory of Insect Biodiversity and Ecosystem Function of Changbai Mountains, Beihua University, Jilin, 132013, China Beihua University Jilin China; 2 School of Forestry, Northeast Forestry University, Harbin, 150040, China Northeast Forestry University Harbin China

**Keywords:** Chalcidoidea, Entedoninae, morphology, new species records, taxonomy

## Abstract

In this paper, four species of *Omphale* Haliday, *O.longigena* Li & Li, **sp. nov.**, *O.longitarsus* Li & Li, **sp. nov.**, *O.rectisulcus* Li & Li, **sp. nov.**, and *O.xanthosoma* Li & Li, **sp. nov.**, are described as new to science; four species, *O.brevibuccata* Szelényi, *O.connectens* Graham, *O.melina* Yefremova & Kriskovich, and *O.obscura* (Förster) are reported from China for the first time; and the male of *O.melina* is reported for the first time in the world. A key to all known species of the genus *Omphale* in China is provided.

## ﻿Introduction

*Omphale* Haliday, 1833 (Hymenoptera, Eulophidae, Entedoninae), containing 271 species worldwide (Noyes 2019; Jamali et al. 2022), is cosmopolitan in distribution and the second largest genus in the Entedoninae. Species of *Omphale* can be separated from other genera in Entedoninae by the following characters: head with frontal sulcus present; interantennal area and antennal scrobe more or less broadened; clypeus distinctly delimited by grooves at least laterally, quadrangular to semicircular, lower margin usually arcuately protruding; occipital median sulcus absent; mesoscutum and mesoscutellum with finely engraved reticulation; propodeum smooth; males usually with verticillate setae on flagellomeres.

The genus *Omphale* from America and Europe are well studied. Two-hundred three (203) species from America and Europe were divided into 18 species groups by Hansson (1996, 1997, 2004) and Hansson and Shevtsova (2012), respectively. Keys to Nearctic, Mexican, Costa Rican and European species were also given. Before this study, there were only eleven *Omphale* species known from China: two species (*O.longiventris* (Ling, 1994) and *O.pulchra* (Ling, 1994)) were described by Ling (1994); nine species (*O.gibsoni* Hansson, 2004, *O.longiseta* Hansson, 1996, *O.masneri* Hansson, 1996, *O.mellea* Hansson, 1996, *O.salicis* (Haliday, 1833), *O.stelteri* (Boucek, 1971), *O.straminea* Hansson, 1996, *O.sulciscuta* (Thomson, 1878), and *O.theana* (Walker, 1839)) were reported by Zhu and Huang (2001, 2002) and Zhang et al. (2007) during taxonomic studies on Eulophidae from Zhejiang, Guangxi, and Gansu provinces of China, respectively. Since then, there have been no further reports of *Omphale* from China.

This paper includes eight additional species, of which four, *O.longigena* sp. nov., *O.longitarsus* sp. nov., *O.rectisulcus* sp. nov., and *O.xanthosoma* sp. nov. are described as new to science; four species, *O.brevibuccata* Szelényi, *O.connectens* Graham, *O.melina* Yefremova & Kriskovich, and *O.obscura* (Förster) are recorded from China for the first time, and the male of *O.melina* is reported for the first time in the world. The species *O.sulciscuta* (Thomson) has new distribution data for China. Detailed descriptions and illustrations of the new species, diagnoses and illustrations of the five previously described species, and a key to all known species of *Omphale* in China are given.

## ﻿Materials and methods

Specimens were collected by sweep nets, yellow-pan traps, and Malaise traps, and were mounted on triangular cards or in Canada Balsam on slides after dissection following methods described by Noyes (1982). Photographs were taken with an Aosvi AO-HK830-5870T digital microscope or a digital CCD camera attached to an Olympus BX51 compound microscope. The quality of these images was improved by using Helicon Focus 7 and Adobe Photoshop 2022. Measurements were made using the built-in software of Aosvi AO-HK830-5870T.

Terminology follows the Hymenoptera Anatomy Consortium (2024), and the following abbreviations are used:

**F1–5** flagellomeres 1–5;

**HE** height of eye;

**MS** malar space;

**MV** marginal vein;

**OOL** minimum distance between a posterior ocellus and corresponding eye margin;

**PMV** postmarginal vein;

**POL** minimum distance between posterior ocelli;

**SMV** submarginal vein;

**STV** stigmal vein;

**WM** width of mouth opening.

Type material is deposited in the insect collections at Northeast Forestry University (**NEFU**), Harbin, China. Abbreviations for other depositories:

**HDOU** Hope Department, Oxford University, Oxford, England

**HNHM**Hungarian Natural History Museum, Budapest, Hungary

**LUZN**Zoological Museum, Lund University, Sweden

**ZISP**Zoological Institute, St Petersburg, Russia

**NHMV** Naturhistorisches Museum, Vienna, Austria

## ﻿Results

### ﻿Key to Chinese species of *Omphale* (females)

**Table:** 

1	Fore wing with STV enlarged and circular (Fig. 6D); membrane around STV and base of MV infuscate	***O.melina* Yefremova & Kriskovich**
–	Fore wing with STV not enlarged; membrane hyaline or only infuscate below MV	**2**
2	Body mainly yellow without metallic reflections; midlobe of mesoscutum with only 1 pair of setae	**3**
–	Body mainly brown, dark brown to black, with weak or strong metallic reflections; midlobe of mesoscutum with 2 pairs of setae	**4**
3	Mesoscutum and mesoscutellum both with a brown longitudinal stripe along median part (Fig. 10C); fore wing hyaline, without any infuscate part; antenna with flagellum slender, F2 as long as F1	***O.xanthosoma* sp. nov.**
–	Only scutellum occasionally with a median infuscate stripe along median line; fore wing with an infuscate part close to STV; antenna with flagellum stouter, F2 0.8 × as long as F1	***O.mellea* Hansson**
4	Mesoscutellum with a median groove (as in Fig. 9D, E)	**5**
–	Mesoscutellum without median groove	**10**
5	Frontal sulcus straight (Fig. 8A, B); median groove on mesoscutellum shallow and narrow	***O* . *rectisulcus* sp. nov.**
–	Frontal sulcus at least curved slightly, mostly arcuate or V-shaped; median groove on mesoscutellum wide and deep	**6**
6	Mesoscutum with a shallow but distinct median groove in posterior part; PMV at most 0.15 × as long as MV	**7**
–	Median groove on mesoscutum not obvious; PMV long, approximately 0.33 × as long as MV	***O.pulchra* (Ling)**
7	Meshes of reticulation on mesoscutellum elongate (Fig. 7D, E)	***O.obscura* (Förster)**
–	Meshes of reticulation on mesoscutellum not elongate in posterior 1/2 at least	**8**
8	Length of body 2.1 mm at least; gaster slender and long, 5.0 × as long as wide	***O.longiventris* (Ling)**
–	Length of body 1.8 mm at most; gaster stouter, 2.0 × as long as wide at most	**9**
9	Antennae with F1 as long as F4, F4 ~ 3.0 × as long as wide (see Bouček, 1971: fig. 13	***O.stelteri* (Bouček)**
–	Antennae with F1 1.15 × as long as F4, and at most 2.2 × as long as wide	***O.sulciscuta* (Thomson)**
10	Fore wing with radial cell bare (as in Fig. 6D)	**11**
–	Fore wing with radial cell setose (as in Fig. 5F)	**13**
11	Clypeus distinctly paler than surrounding parts of face, without metallic reflections	***O* . *straminea* Hansson**
–	Clypeus with more or less metallic reflections and with same color as surrounding parts of face	**12**
12	All coxae dark brown with bluish green metallic reflections; all femora brown	***O.salicis* (Haliday)**
–	All coxae mainly yellowish white, pale brown at base only; all femora yellowish white, metafemur infuscate along dorsal margin	***O.theana* (Walker)**
13	Lateral mesosoma yellowish white to yellowish brown, without metallic reflections	**14**
–	Lateral mesosoma dark brown, with bluish green metallic reflections	**15**
14	Gena elongate, MS 0.4 × as long as HE (Fig. 4A, B); fore wing below MV with a wide infuscate band (Fig. 4I)	***O.longigena* sp. nov.**
–	Gena shorter, MS 0.2 × as long as HE; fore wing hyaline, without infuscate band	***O* . *brevibuccata* Szelényi**
15	Legs with 4^th^ tarsomere on all legs slender and elongate, half as long as whole tarsus (Fig. 5I–K)	***O* . *longitarsus* sp. nov.**
–	Legs with 4^th^ tarsomeres of protarsus and mesotarsus at most 0.4 × as long as the length of corresponding tarsus	**16**
16	Head with frontal cross-ridge present (as in Hansson, 1996: fig. 1)	**17**
–	Head with frontal cross-ridge absent (as in Hansson, 1996: fig. 101)	**18**
17	Setae on vertex and thoracic dorsum distinctly longer than in alternate, outermost seta on vertex as long as POL, hind pair of setae on mesoscutum longer than distance separating them	***O.longiseta* Hansson**
–	Setae on vertex and thoracic dorsum shorter, outermost seta on vertex at most 0.7 × as long as POL, hind pair of setae on mesoscutum at most half the distance separating them	***O.connectens* Graham**
18	Clypeus poorly delimited, more or less semicircular; antenna with scape dark brown except proximal 1/3 pale	***O.masneri* Hansson**
–	Clypeus distinctly delimited, quadrangular; antenna with scape predominantly pale to dark brown with a pale median spot	***O.gibsoni* Hansson**

### ﻿Species descriptions

#### ﻿ Omphale brevibuccata

Taxon classificationAnimaliaHymenopteraEulophidae

Szelényi, 1978

DF4E9CF7-4E8E-5CDE-A35E-AC4A3E2EE563

Figs 1A , 2A–I

Omphale brevibuccata Szelényi, 1978: 222, holotype ♀, Hungary, HNHM, not examined.

##### Material examined.

• 14♀: 1♀ [NEFU; on card, right antenna and right wings on slide], China, Guangdong Province, Shaoguan City, Chebaling National Nature Reserve, 29–30.IV.2019, leg. Wen-Jian Li and Jun Wu, by yellow-pan trapping • 3♀ [NEFU; on cards], China, Shandong Province, Qingdao City, Laoshan Scenic Spot, Beijiushui, 8–10.VII.2014, leg. Hui Geng, Guo-Hao Zu, Zhi-Guang Wu, and Hai-Feng Bai, by yellow-pan trapping • 2♀ [NEFU; on cards], China, Shandong Province, Qingdao City, Jimo District, Mashan Park, 11.VII.2014, leg. Si-Zhu Liu, Guo-Hao Zu, and Zhi-Guang Wu, by sweep netting • 2♀ [NEFU; 1 on slide, 1 on card], China, Shandong Province, Qingdao City, Laoshan Scenic Spot, Beijiushui, 12.VII.2014, leg. Hui Geng, Guo-Hao Zu, Zhi-Guang Wu, and Hai-Feng Bai, by sweep netting • 4♀ [NEFU; on cards], China, Shandong Province, Qingdao City, Dazhushan, 13–14.VII.2014, leg. Ye Chen and Chao Zhang, by yellow-pan trapping • 2♀ [NEFU; on cards], China, Liaoning Province, Anshan City, Qianshan, 20.VIII.2015, leg. Hui Geng, Yan Gao, and Zhi-Guang Wu, by sweep netting.

##### Diagnosis.

**Female.** Body length 1.2–1.8 mm. Vertex golden-green, face dark brown with golden or purple reflections; antenna with scape yellow to pale brown, pedicel and flagellum brown; mesoscutum dark brown, mesoscutellum with anterior 1/3–1/2 yellowish brown and remainder brown to dark brown; axillae with anterior 1/2 dark brown to brown, remainder yellowish brown to yellowish white; propodeum pale brown; other parts of mesosoma yellow to yellowish white (including legs); gaster dark brown. Head (Fig. 2A) with antennal scrobes meeting on the V-shaped frontal sulcus; frontal cross-ridge absent; gena very short, HE:MS:WM ~ 5.0:1.0:3.4; clypeus semicircular 1.7 × as wide as high; antenna (Fig. 2B) slender, with five flagellomeres separated from each other, F1 1.1 × as long as F2. Mesosoma (Fig. 2C) with mesoscutum and scutellum with weak reticulation; midlobe of mesoscutum with two pairs of setae; scutellum 1.0–1.2 × as long as wide, with anterior margin almost straight; propodeum smooth, with a weak median carina, and anteromedially with a fovea. Fore wing (Fig. 2D) speculum closed, with seven or eight admarginal setae arising from MV and membrane just behind MV, radial cell setose, PMV 1.8 × as long as STV. Metasoma (Fig. 2F), gaster 1.6 × as long as mesosoma, and nearly as long as head + mesosoma.

**Figure 1.**
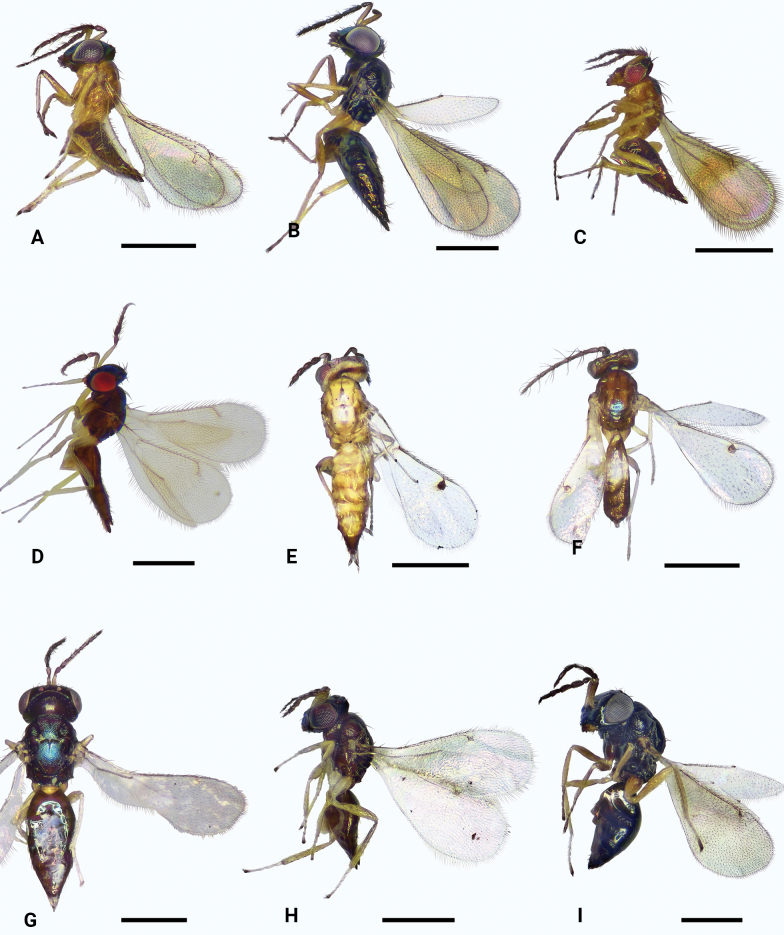
*Omphale* spp., habitus **A***O.brevibuccata*, ♀, lateral **B***O.connectens*, ♀, lateral **C***O.longigena* sp. nov., holotype, ♀, lateral **D***O.longitarsus* sp. nov., holotype, ♀, lateral **E***O.melina*, ♀, dorsal **F***O.melina*, ♂, dorsal **G***O.rectisulcus* sp. nov., holotype, ♀, dorsal **H***O.obscura*, ♀, lateral **I***O.sulciscuta*, ♀, lateral. Scale bars: 500 μm.

**Figure 2.**
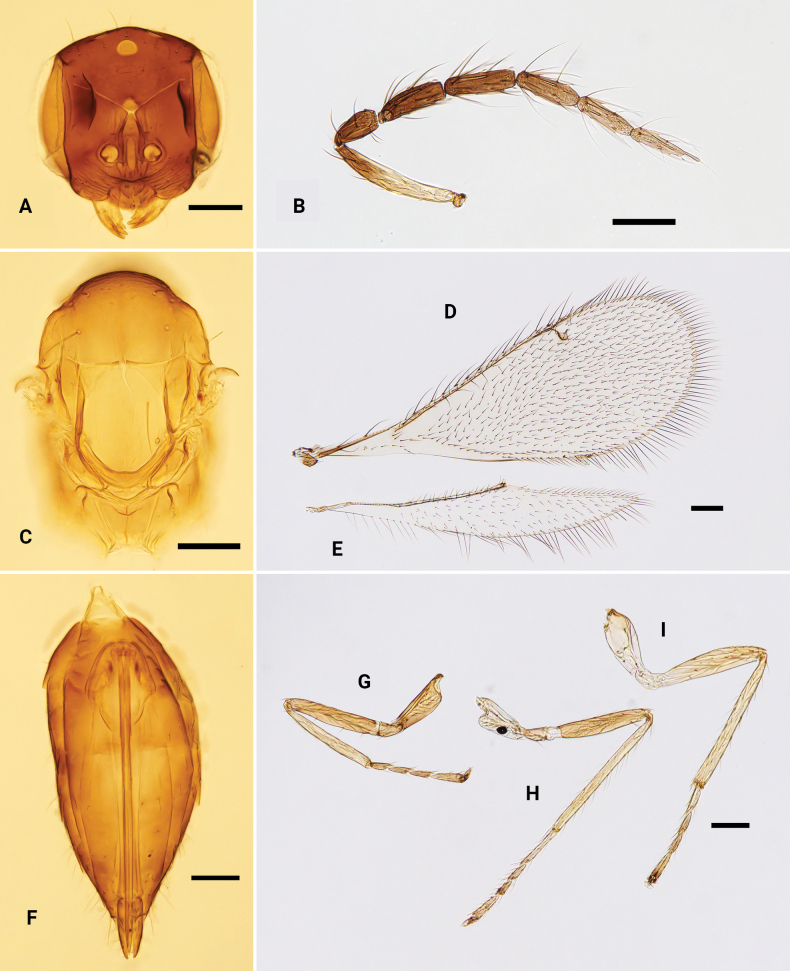
*Omphalebrevibuccata*, ♀ **A** head, frontal view **B** antenna **C** mesosoma, dorsal view **D** fore wing **E** hind wing **F** metasoma, dorsal view **G–I** fore, mid and hind leg, respectively. Scale bars: 100 μm.

**Male.** Unknown.

##### Host.

Unknown.

##### Distribution.

China (Liaoning, Shandong, and Guangdong Provinces) (new record); Hungary (Szelényi 1978); United Kingdom (Askew 2003); Russia, Sweden, Netherlands, Bulgaria (Hansson and Shevtsova 2012); Romania (Hansson 2016).

#### ﻿ Omphale connectens

Taxon classificationAnimaliaHymenopteraEulophidae

Graham, 1963

CAB32825-30FE-5D9C-BA8C-396CF102BBFA

Figs 1B , 3A–K

Omphale connectens Graham, 1963: 261, holotype ♀, Berkshire, England, UK, HDOU, not examined.

##### Material examined.

• 4♀: 2♀ [NEFU; 1 on card, 1 on slide], China, Chongqing City, Simian Mountain, Dawopu, 04.VIII.2018, leg. Guang-Xin Wang and Jun-Jie Fan, by sweep netting • 2♀ [NEFU; 1 on card, 1 on slide], China, Inner Mongolia, Ulanhot City, Sanhe Village, 12.VII.2021, leg. Yuan-Yuan Jin and Yue Qin, by sweep netting.

##### Diagnosis.

**Figure 3.**
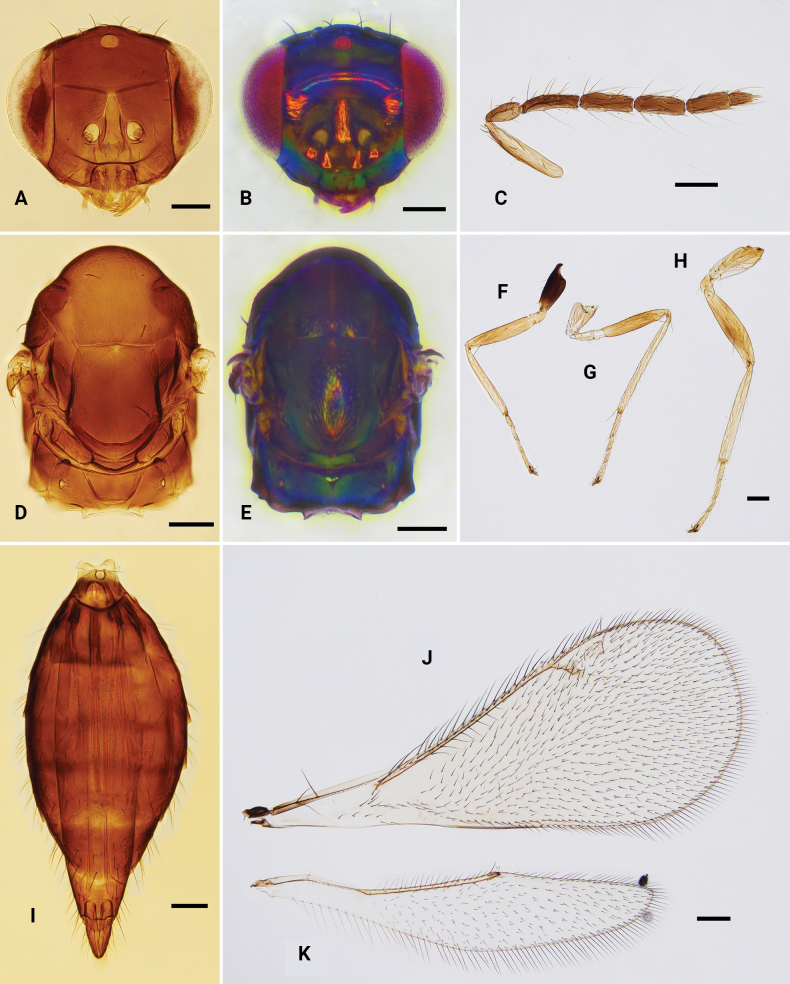
*Omphaleconnectens*, ♀ **A, B** head, frontal view **C** antenna **D, E** mesosoma, dorsal view **F–H** fore, mid and hind leg, respectively **I** metasoma, dorsal view **J** fore wing **K** hind wing. Scale bars: 100 μm.

**Female.** Body length 1.0–2.4 mm. Vertex and face bronze, golden green, golden blue to purple metallic; antenna with scape yellow, along dorsal edge dark brown, pedicel and flagellum dark brown; mesoscutum bluish green, bronze to purple metallic; scutellum with similar color to mesoscutum, sometime darker; propodeum bluish green to purple metallic; legs with procoxa dark brown, mesocoxa and metacoxa yellowish brown to yellow; femora yellowish brown to brown; tibiae yellow to yellowish brown; protarsus pale brown, mesotarsus and metatarsus yellow to yellowish brown; gaster with first tergite bluish green metallic, remainder dark brown metallic. Head (Fig. 3A, B) with face and vertex smooth to with very weak sculpture partly; antennal scrobes meeting on the nearly V-shaped frontal sulcus; frontal cross-ridge present; clypeus trapezoid to semicircular 1.4–1.6 × as wide as high; antenna (Fig. 3C) with 3-segmented funicle and 2-segmented clava, F1–3 with two sets of setae, one set attached closed to base and another attached subapically or medially on the flagellomere, F1 1.1 × as long as F2. Mesosoma (Fig. 3D, E) with mesoscutum and scutellum with shallow reticulation; midlobe of mesoscutum with two pairs of setae; scutellum 1.2 × as long as wide, with anterior margin almost straight or weakly curved forwards; propodeum smooth, without median carina. Fore wing (Fig. 3J) speculum closed, with 6–10 admarginal setae arising from MV and membrane just below MV, STV short, PMV 1.5–2.0 × as long as STV, radial cell small and setose, only along PMV bare. Metasoma (Fig. 3I), gaster 1.6–2.0 × as long as mesosoma, and longer than head + mesosoma (1.2:1.0).

**Male.** Not collected from China, see Hansson and Shevtsova (2012).

##### Host.

Unknown.

##### Distribution.

China (Inner Mongolia and Chongqing Provinces) (new record); United Kingdom (Graham 1963); Ireland (north and south) (Bouček and Askew 1968); France, Germany, Netherlands (Gijswijt 1976); Croatia, Serbia (Bouček 1977); Sweden (Hansson 1991); Czech Republic, Denmark, Hungary, Russia (Hansson and Shevtsova 2012); Romania (Hansson 2016).

#### ﻿ Omphale longigena

Taxon classificationAnimaliaHymenopteraEulophidae

Li & Li sp. nov.

29D35BA7-F797-5BA4-B5FD-DB007E057987

https://zoobank.org/4EEBF025-FB50-481A-AD26-E2BB7A85168F

Figs 1C , 4A–J

##### Material examined.

***Holotype***: • ♀ [NEFU; on card], China, Shandong Province, Qingdao City, Jimo District, Mashan Park, 11.VII.2014, leg. Si-Zhu Liu, Guo-Hao Zu, and Zhi-Guang Wu, by sweep netting. ***Paratypes***: • 12♀: 1♀ [NEFU; on slide], same data as the holotype • 2♀ [NEFU; on cards], China, Shandong Province, Qingdao City, Dahedong Village, 10.VII.2014, leg. Si-Zhu Liu, Ye Chen and Chao Zhang, by sweep netting • 3♀ [NEFU; on cards], China, Shandong Province, Qingdao City, Laoshan Scenic Spot, Beijiushui, 8–10.VII.2014, leg. Hui Geng, Guo-Hao Zu, Zhi-Guang Wu, and Hai-Feng Bai, by yellow-pan trapping • 2♀ [NEFU; 1 on card, 1 on slide], China, Guangdong Province, Shaoguan City, Chebaling National Nature Reserve, 29–30.IV.2019, leg. Wen-Jian Li and Jun Wu, by yellow-pan trapping • 4♀ [NEFU; on cards], China, Guangdong Province, Shaoguan City, Chebaling National Nature Reserve, 1–2.V.2019, leg. Wen-Jian Li and Jun Wu, by yellow-pan trapping.

##### Diagnosis.

**Female.** Frontal sulcus slightly V-shaped; antennal scrobes meeting on frontal sulcus; antennal toruli situated completely below the level of lower eye margin; gena distinctly elongate, MS 0.4 × as long as HE; fore wing with a broad infuscate band below MV beyond speculum, extending to STV and posterior margin of wing; admarginal setae seven or eight, arising from both MV and membrane just below MV, and the most apical seta attached close to STV; PMV slightly shorter than STV.

##### Description.

**Female.** Body length 1.0–1.2 mm. Upper face and vertex brown to dark brown with golden-green reflections, lower face yellowish brown, eyes dull red, clypeus with same color as surrounding parts of face, mandibles yellowish white with apex brown. Mesosoma with mid lobe of mesoscutum golden-green, mesoscutellum dark brown, remaining parts of mesosoma yellow to pale brown. Metasoma brown to dark brown except yellow petiole. Antenna with scape yellowish brown with dorsal margin dark brown, pedicel and flagellum brown to dark brown. All legs yellow, except dark brown tarsal claws. Fore wing with a broad infuscate band below MV beyond speculum, extending to STV and to posterior margin of wing.

**Figure 4.**
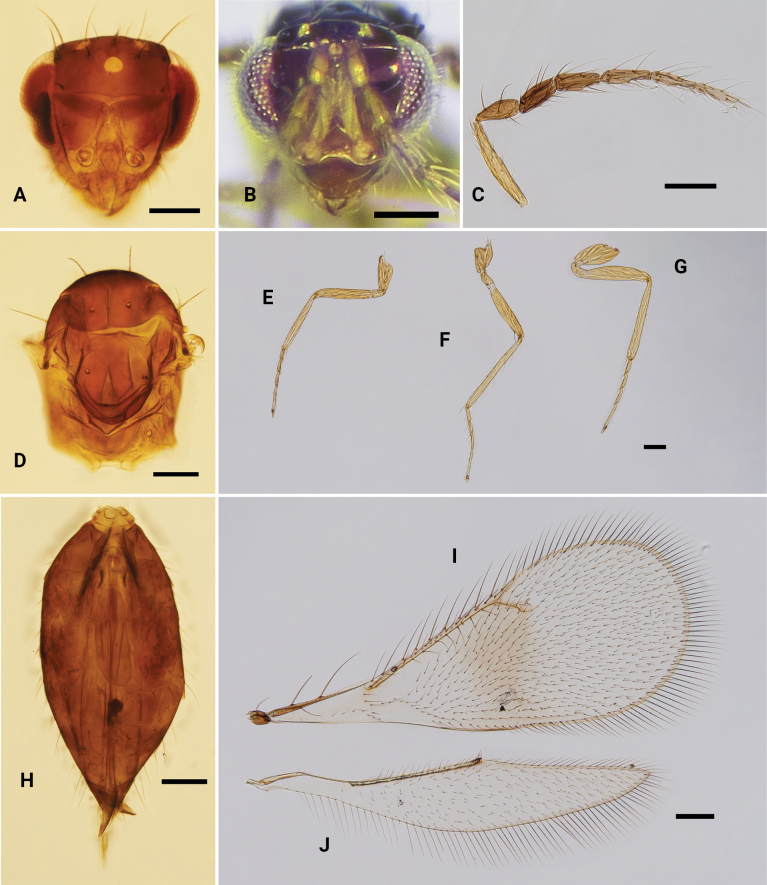
*Omphalelongigena* sp. nov., paratype, ♀ **A, B** head, frontal view **C** antenna **D** mesosoma, dorsal view **E–G** fore, mid and hind leg, respectively **H** metasoma, dorsal view **I** fore wing **J** hind wing. Scale bars: 100 μm.

***Head*** (Fig. 4A, B) in frontal view 1.2 × as wide as high, usually collapsed after death; face and vertex smooth. POL:OOL ~ 1.5:1.0; frontal sulcus slightly V-shaped, reaching eye margin, the midpoint closer to median ocellus than to antennal toruli; antennal scrobes meeting on frontal sulcus; antennal toruli situated completely below the level of lower eye margin; frontal cross-ridge present, gena distinctly elongate, HE:MS:WM ~2.6:1.0:1.8; clypeus weakly delimited with lower margin distinctly protruding, 1.8–1.9 × as wide as high; mandible with two large teeth at apex and a row of smaller teeth at base. Antenna (Fig. 4C) slender, with all five flagellomeres separated from each other; scape 6.3 × as long as wide; pedicel 2.2 × as long as wide, and as long as F1; flagellomeres decreasing in width from F1 to F5, F1 1.0 × as long and 1.1 × as wide as F2.

***Mesosoma*** (Fig. 4D) 1.4 × as long as wide; pronotum reduced and not visible in dorsal view; mesoscutum, mesoscutellum, metascutellum and propodeum smooth, without any sculpture; mid lobe of mesoscutum with two pairs of setae; notauli indicated only in anterior 1/3; mesoscutellum 1.2 × as long as wide, with one pair of setae located in median part; metascutellum smooth, nearly triangular, 0.4 × as long as wide, and 0.5 × as long as length of median propodeum; propodeum without median carina. Fore wing (Fig. 4I) 2.5 × as long as wide, speculum closed; with seven or eight admarginal setae arising from both MV and from membrane just below MV, with apical setae attached close to STV; PMV slightly shorter than STV, radial cell setose, ratio of SMV:MV:PMV:STV ~ 3.8:4.9:1.0:1.3. Hind wing (Fig. 4J) 5.4 × as long as wide, apex pointed. All legs (Fig. 4E–G) with 4^th^ tarsomere slightly elongate, 0.3–0.4 × as long as whole tarsus; metatibial spur short, only reaching the middle of 1^st^ tarsomere.

***Metasoma*** (Fig. 4H) 2.0 × as long as wide; petiole short; gaster 1.4 × as long as length of mesosoma, and longer than head + mesosoma (1.2:1.0); ovipositor sheaths exserted beyond apex of gaster.

**Male.** Unknown.

##### Host.

Unknown.

##### Etymology.

The specific name refers to the elongate gena.

##### Distribution.

China (Shandong and Guangdong Provinces).

##### Remarks.

*Omphalelongigena* sp. nov. is very similar to *O.litera* Jamali & Zeya, 2022. The two species share the following characteristics: head with gena elongate, MS 0.4 × as long as HE; flagellomeres decreasing in width from F1 to F5; fore wing with a broad infuscate band below MV beyond speculum, extending to STV and to posterior margin of disc; mesoscutum, mesoscutellum, metascutellum and propodeum smooth. *Omphalelongigena* sp. nov. differs from *O.litera* in having the antennal torulus situated completely below the level of lower eye margin (vs above lower eye margin in *O.litera*); scape 6.3 × as long as wide (vs 4.2 × in *O.litera*), hind leg yellow, except dark brown tarsal claws (vs hind leg with coxa, femur basally three fourths and last tarsomere brown in *O.litera*). The figure in Jamali et al. (2022: fig. 2C) shows that the scape of the holotype of *O.litera* was damaged, and the color of the leg usually has variation, so the latter two differences may not be reliable characters for identification. The most reliable characteristic to distinguish these two species is the position of the antennal torulus.

#### ﻿ Omphale longitarsus

Taxon classificationAnimaliaHymenopteraEulophidae

Li & Li sp. nov.

76523B1B-CE49-5EB0-A640-AF6CFB89380A

https://zoobank.org/5A68F1ED-E168-479F-AAC0-F8378FA6EAEF

Figs 1D , 5A–K

##### Type material.

***Holotype***: • ♀ [NEFU; on card], China, Xizang Autonomous Region, Medog County, Damu Village, 22–29.VI.2017, leg. Zhaxi, by Malaise trapping. ***Paratypes***: • 2♀: 1♀ [NEFU; on slide], same data as the holotype • 1♀ [NEFU; on card], China, Xizang Autonomous Region, Medog County, Gedang Village, 31.V–5.VI.2021, leg. Jun-Jie Fan and Jun Wu, by yellow-pan trapping.

##### Diagnosis.

**Female.** Frontal sulcus slightly curved, nearly straight, reaching eye margin; clypeus quadrangular with lower margin arcuately protruding, 1.9–2.0 × as wide as high; antenna slender, flagellomeres decreasing in width from F1 to F5; propodeum smooth and flat, with a narrow groove along anterior margin, without median carina; all legs with apical tarsomere slander and elongate, nearly as long as half the length of whole tarsus.

##### Description.

**Female.** Body length 1.4–1.5 mm. Face and vertex bronze with golden-green reflections, eyes red, clypeus with same color as surrounding parts of face, mandibles yellowish white with base and apex brown. Mesosoma brown with weak golden-blue or golden-green reflections. Metasoma brown to dark brown, except yellow petiole. Antenna with scape yellowish white, pedicel and flagellum brown, gradually lighten towards apex, F5 yellowish white. All legs yellowish white except brown claws and fore coxae. Fore wings hyaline.

**Figure 5.**
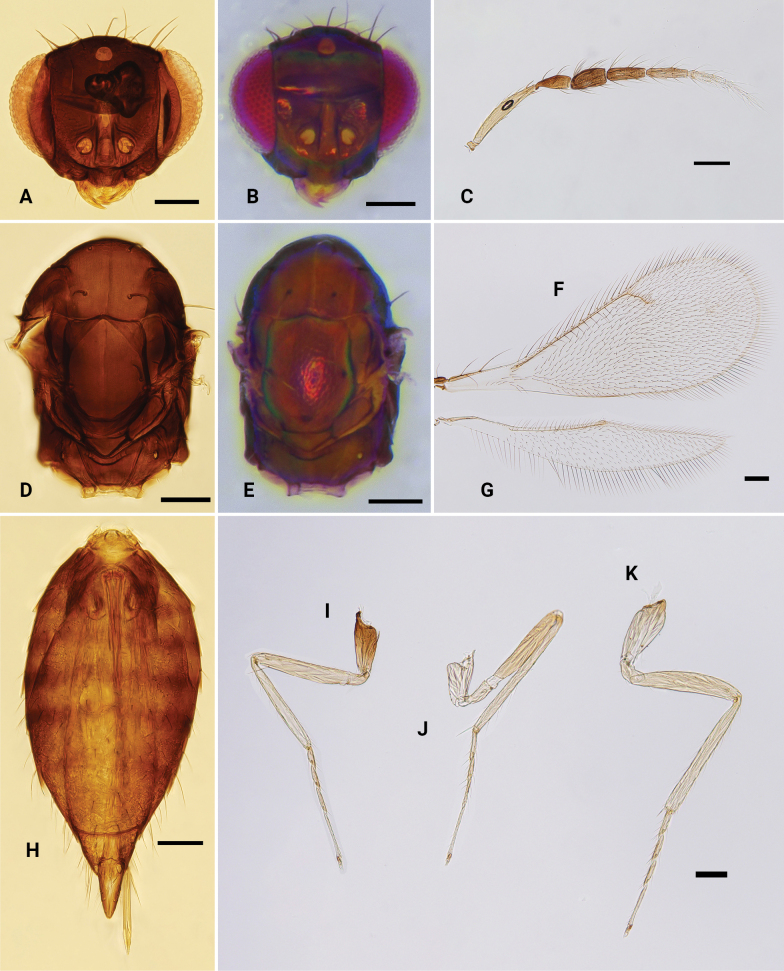
*Omphalelongitarsus* sp. nov., paratype, ♀ **A, B** head, frontal view **C** antenna **D, E** mesosoma, dorsal view **F** fore wing **G** hind wing **H** metasoma, dorsal view **I–K** fore, mid and hind leg, respectively. Scale bars: 100 μm.

***Head*** (Fig. 5A, B) in frontal view 1.3 × as wide as high. Face between frontal sulcus and frontal cross-ridge with weak and irregular sculpture, remainder of face and vertex smooth; POL:OOL ~ 1.8:1.0; frontal sulcus slightly curved, nearly straight, reaching eye margin, the midpoint closer to antennal torulus than median ocellus; antennal scrobes join frontal sulcus separately; subtorular grooves and frontal cross-ridge present; clypeus quadrangular with lower margin arcuately protruding, 1.9–2.0 × as wide as high; mandible with two large teeth at apex and a row of smaller teeth at base; HE:MS:WM ~ 2.8:1.0:1.2. Antenna (Fig. 5C) slender, with all five flagellomeres separated from each other; scape 6.5 × as long as wide; pedicel 2.4 × as long as wide, and 0.6 × as long as F1; flagellomeres decreasing in width from F1 to F5, F1 0.9 × as long and 1.5 × as wide as F2.

***Mesosoma*** (Fig. 5D, E) 1.5 × as long as wide; pronotum reduced and not visible in dorsal view; mesoscutum smooth and flat, with two pairs of setae; notauli indicated only in anterior third; mesoscutellum flat, with very weak traces of reticulation, 1.4 × as long as wide, with one pair of setae located in the middle and close to lateral margin; axillae smooth; metascutellum smooth, nearly triangular, 0.4 × as long as wide, and 0.5 × as long as length of median propodeum. Lateral panels of metanotum smooth; propodeum smooth and flat, with a narrow groove along anterior margin, without median carina. Fore wing (Fig. 5F) 2.6 × as long as wide, with rather dense setae on membrane, speculum closed; with nine admarginal setae arising from MV; PMV shorter than STV, radial cell setose, ratio of SMV:MV:PMV:STV ~ 4.3:7.8:1.0:1.8. Hind wing (Fig. 5G) 5.5 × as long as wide, apex slightly pointed. All legs (Fig. 5I–K) with apical tarsomere slander and elongate, nearly half the length of whole tarsus; metatibial spur distinctly shorter than basal tarsomere, only reaching the middle of basal tarsomere.

***Metasoma*** (Fig. 5H) 2.2 × as long as wide; petiole short; gaster 1.6 × as long as the length of mesosoma, and longer than head + mesosoma (1.2:1.0); ovipositor sheaths exserted beyond apex of gaster.

**Male.** Unknown.

##### Host.

Unknown.

##### Etymology.

The specific name refers to the elongate tarsus.

##### Distribution.

China (Xizang Autonomous Region).

##### Remarks.

*Omphalelongitarsus* sp. nov. should belong to *Aetius* group, and can be separated from other species by having antenna slender, flagellomeres decreasing in width distinctly from F1 to F5, F1 0.9 × as long and 1.5 × as wide as F2; all legs with apical tarsomere slander and elongate, nearly half the length of whole tarsus.

#### ﻿ Omphale melina

Taxon classificationAnimaliaHymenopteraEulophidae

Yefremova & Kriskovich, 1994

3170795F-A4E5-5102-949D-156A1F6EB611

Figs 1E, F , 6A–G

Omphale melinum Yefremova & Kriskovich, 1994: 247, holotype ♀, Russia-Primorsky Krai, ZISP, not examined.
Omphale melina Yefremova & Kriskovich: Hansson and Shevtsova (2012): 139.

##### Material examined.

• 6♀1♂: 2♀1♂ [NEFU; on cards, right antenna of ♂ on slide], China, Liaoning Province, Fushun City, Dahuofang Forestry Station, 18.VI.2012, leg. Hui Geng, Xiang-Xiang Jin, and Jiang Liu, by sweep netting • 1♀ [NEFU; on card], China, Liaoning Province, Fushun City, Yuanshuailin (Marshal Mausoleum), 18.VI.2012, leg. Hui Geng, Xiang-Xiang Jin, and Jiang Liu, by sweep netting • 1♀ [NEFU; on card], China, Liaoning Province, Anshan City, Qianshan, 21.VI.2015, leg. Hui Geng, Si-Zhu Liu, Yan Gao, and Zhi-Guang Wu, by sweep netting • 2♀ [NEFU; on cards], China, Liaoning Province, Anshan City, Qianshan, 23.VI.2015, leg. Hui Geng, Si-Zhu Liu, Yan Gao, and Zhi-Guang Wu, by sweep netting.

##### Diagnosis.

**Figure 6.**
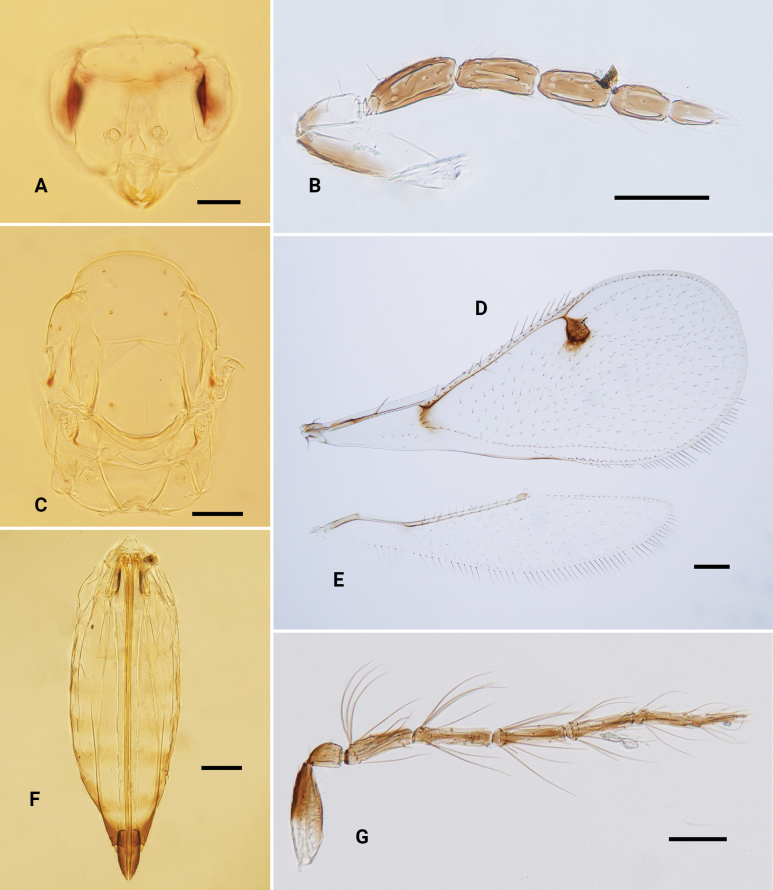
*Omphalemelina***A** head, frontal view, ♀ **B** antenna, ♀ **C** mesosoma, dorsal view, ♀ **D** fore wing, ♀ **E** hind wing, ♀ **F** metasoma, dorsal view, ♀ **G** antenna, ♂. Scale bars: 100 μm.

**Female.** Body length 1.2–1.5 mm, mainly yellowish white, yellow to pale brown without metallic reflection; occiput with a brown transverse stripe; along median line of mesoscutum and scutellum dark brown; 7^th^ tergite and apical parts of ovipositor sheaths dark brown to black; antenna with scape yellowish white, only dorsal part dark brown; pedicel yellowish white, brown at base; flagellum dark brown; fore wing hyaline, with infuscate around STV and base of MV; legs mainly yellow to yellowish white. Head (Fig. 6A) with face with weak sculpture; frontal sulcus nearly straight, antennal scrobes meeting on frontal sulcus; frontal cross-ridge absent; clypeus semicircular 1.3–1.4 × as wide as high; mandible tridentate. Antenna (Fig. 6B) with five flagellomeres separated from each other; scape slightly stout, 3.4 × as long as wide; pedicel 2.0 × as long as wide; flagellomeres decreasing in length distinctly from F1 to F5, F1 1.2 × as long and 1.1 × as wide as F2. Mesosoma (Fig. 6C) with mesoscutum and scutellum with shallow reticulation; midlobe of mesoscutum with two pairs of setae; scutellum 1.2 × as long as wide; propodeum smooth, without median carina. Fore wing (Fig. 6D) speculum closed; with 4–5 admarginal setae arising from MV and membrane just below MV; STV enlarged and subcircular, PMV 1.0–1.2 × as long as STV, radial cell bare. Metasoma (Fig. 6F), gaster elongate, 1.7 × as long as mesosoma, longer than head + mesosoma (1.4:1.0).

**Male.** Body length 1.2 mm, mainly brown and with weak metallic reflection; antenna brown, only basal 1/3 of scape yellowish white. Antenna (Fig. 6G) slender and longer than female, F1–4 with verticillate setae and the setae reaching beyond apex of flagellomere attached to; F1 0.9 × as long and 1.2 × as wide as F2. Gaster with an oval and transparent membranous region between 1^st^ tergite and 4^th^ tergite. Other features as in female.

##### Host.

Unknown.

##### Distribution.

China (Liaoning Province) (new record); Russia (Yefremova and Kriskovich 1994).

##### Remarks.

The male of *Omphalemelina* is recorded for the first time in the world. The color of females collected from China is distinctly lighter than in the paratype from Russia, while the male collected from China has similar color to the paratype female from Russia (Hansson and Shevtsova (2012: figs 468–470). Both sexes of *O.melina* can be easily separated from other species in *Omphale* through its enlarged stigmal vein and non-metallic body.

#### ﻿ Omphale obscura

Taxon classificationAnimaliaHymenopteraEulophidae

(Förster, 1841)

C1FCBEB3-E81B-5C33-A0F8-0E81A4E172A8

Figs 1H , 7A–K

Elachestus obscurus Förster, 1841: 40, lectotype ♀, Germany, NHMV, not examined.
Holcopelte obscura (Förster): Förster, 1856: 81.
Holcopelte fulvipes Förster, 1861: 137, lectotype ♀, Switzerland, NHMV, not examined.
Horismenus obscurus (Förster): Schmiedeknecht 1909: 433.
Horismenus fulvipes (Förster): Schmiedeknecht 1909: 433.
Omphale obscura (Förster): Hansson and Shevtsova 2012: 113.

##### Material examined.

• 12♀: 4♀ [NEFU; 3 on cards, 1 on slide], China, Liaoning Province, Anshan City, Qianshan, 21.VI.2015, leg. Hui Geng, Si-Zhu Liu, Yan Gao, and Zhi-Guang Wu, by sweep netting • 3♀ [NEFU; on cards], China, Liaoning Province, Huludao City, Jianchang county, Bailangshan National Nature Reserve, 04.VII.2012, leg. Si-Zhu Liu and Jiang Liu, by sweep netting • 2♀ [NEFU; 1 on card, 1 on slide], China, Xizang Autonomous Region, Medog County, Damu Village, 22–29.VI.2017, leg. Zhaxi, by Malaise trapping • 2♀ [NEFU; on cards], China, Xizang Autonomous Region, Medog County, Damu Village, 15–22.VI.2017, leg. Zhaxi, by Malaise trapping • 1♀ [NEFU; on card], China, Xizang Autonomous Region, Medog County, Gedang Village, 31.V–05.VI.2021, leg. Jun-Jie Fan and Jun Wu, by yellow-pan trapping.

##### Diagnosis.

**Figure 7.**
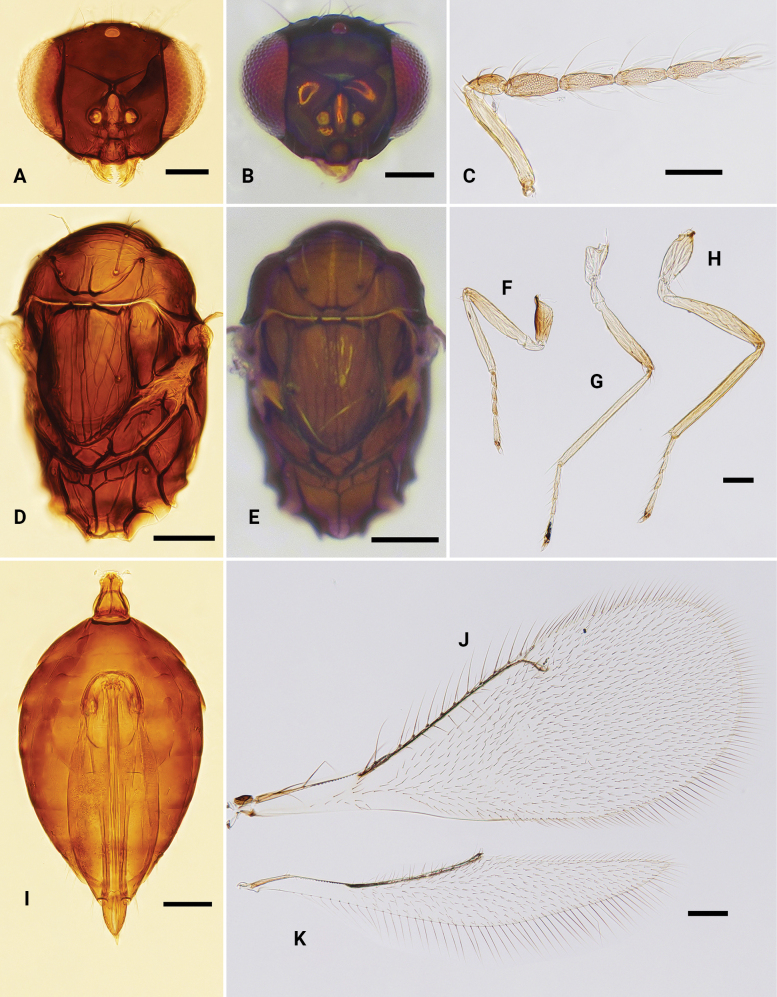
*Omphaleobscura*, ♀ **A, B** head, frontal view **C** antenna **D, E** mesosoma, dorsal view **F–H** fore, mid and hind leg, respectively **I** metasoma, dorsal view **J** fore wing **K** hind wing. Scale bars: 100 μm.

**Female.** Body length 1.2–1.4 mm, mainly brown to dark brown, face and vertex with bronze metallic tinges; eyes dull red; antenna with scape yellow to pale brown, pedicel and flagellum brown to dark brown; mandibles pale brown to yellow; legs yellow to yellowish white, except dark brown claws and brown fore coxa; wings hyaline. Head (Fig. 7A, B) not collapsed after death; face and vertex smooth, without any reticulation; POL:OOL ~ 1.3:1.0; frontal sulcus V-shaped, reaching eye margin, the midpoint closer to antennal toruli than to median ocellus; antennal scrobes meeting just below frontal sulcus and connected to frontal sulcus by a short longitudinal suture; HE:MS:WM ~ 3.9:1.0:2.3; clypeus trapezoid to triangular, as high as the width at its lower margin, lower margin protruding and emarginate; mandible with two large and pointed teeth at apex and 3–4 smaller and obtuse teeth at base. Antenna (Fig. 7C) with all five flagellomeres separated from each other; scape 5.3 × as long as wide, longer than pedicel + F1; pedicel 1.8 × as long as wide; F1–5 with verticillate setae, setae on F1 reaching apex of F1, and setae on F2–5 reaching beyond apex of flagellomere attached to; F1 1.0 × as long and 1.4 × as wide as F2; F3 to F5 slightly decreasing in both length and width. Mesosoma (Fig. 7D, E), 1.6 × as long as wide; mesoscutum with sparse reticulation with transverse meshes, mid lobe with two pairs of setae, with a shallow median groove in posterior 1/3; notauli as subtriangular impressions in posterior 1/2; mesoscutellum 1.3 × as long as wide, with sparse and elongate reticulation or striation and one pair of setae, anterior 1/2 with an indistinct and shallow median groove; metascutellum with two foveae anterolaterally, 0.4 × as long as wide, 0.3 × as long as length of median propodeum; propodeum 0.5 × as long as mesoscutellum, smooth, with a distinct and complete median carina and a pair of plicae, as well as a wide groove along anterior margin, posteromedian part forms a short nucha. Fore wing (Fig. 7J) 2.8 × as long as wide, speculum closed; with six to ten admarginal setae arising from both MV and from membrane just below MV; STV long and slender, PMV shorter than STV, radial cell setose, ratio of SMV:MV:PMV:STV ~ 5.9:10.0:1.0:1.5. Hind wing (Fig. 7K) 6.3 × as long as wide, apex pointed. Legs (Fig. 7F–H) with metatibial spur very short, not reaching the middle of basal tarsomere. Metasoma (Fig. 7I) with petiole pyriform, 0.6 × as long as length of median propodeum; gaster 1.2 × as long as length of mesosoma, nearly as long as head + mesosoma; ovipositor sheaths exserted beyond apex of gaster.

**Male.** Not collected from China, see Hansson and Shevtsova (2012).

##### Host.

*Dasineuraviciae* (Diptera, Cecidomyiidae) (Bouček and Askew 1968).

##### Distribution.

China (Xizang Autonomous Region and Liaoning Province) (new record); Germany (Förster 1841); Switzerland (Förster 1861); Hungary (Erdös 1956); Austria, Sweden, United Kingdom, Yugoslavia (Bouček and Askew 1968); Croatia (Bouček 1977); Czech Republic (Kalina 1989); Italy (De Stefani 1905); Netherlands (Gijswijt 2003); France, Russia (Hansson and Shevtsova 2012); Romania (Hansson 2016).

##### Remarks.

The specimens collected from China have distinct propodeal plicae that almost reach the anterior margin of the propodeum, whereas the European specimens have irregular plicae that only reach half the length of the propodeum (Hansson and Shevtsova 2012: fig. 376).

#### ﻿ Omphale rectisulcus

Taxon classificationAnimaliaHymenopteraEulophidae

Li & Li sp. nov.

8A836BF6-A3A7-537A-8137-BD94CF5951F2

https://zoobank.org/4DC8D2ED-A242-4974-8A63-3FFF470CDC8F

Figs 1G , 8A–K

##### Type material.

***Holotype***: • ♀ [NEFU; on card], China, Jiangxi Province, Shangrao City, Yanshan County, Yejiachang Village, 7.VII.2013, leg. Chao Zhang, by sweep netting. ***Paratypes***: • 1♀ [NEFU; on slide], China, Sichuan Province, Guangyuan City, Qingchuan County, 20.VIII.2015, leg. Ye Chen and Chao Zhang, by sweep netting.

##### Diagnosis.

**Female.** Frontal sulcus straight, reaching eye margin, distance from frontal sulcus to median ocellus is as long as distance to toruli; antennal scrobes slightly wide and deep, as grooves rather than sulcus, meeting below frontal sulcus and connected to frontal sulcus by a short longitudinal suture; clypeus with both upper and lower margins arcuate, nearly oval-shaped, 2.0 × as wide as high; notauli step-shaped, indicated by smooth depressions; propodeum with a narrow groove along anterior margin, without median carina.

##### Description.

**Female.** Body length 1.5–1.6 mm. Face and vertex dark brown with weak golden-green and bronze reflections; eyes dull red; clypeus with same color as surrounding parts of face; mandibles yellowish white with apex brown; mesosoma brown with golden-blue and golden-green reflections; metasoma brown to dark brown except yellow petiole; antenna with scape yellowish white, pedicel and flagellum brown; all legs yellowish white except fore coxae and fore tarsi, which are brown or yellowish brown; fore wings hyaline.

**Figure 8.**
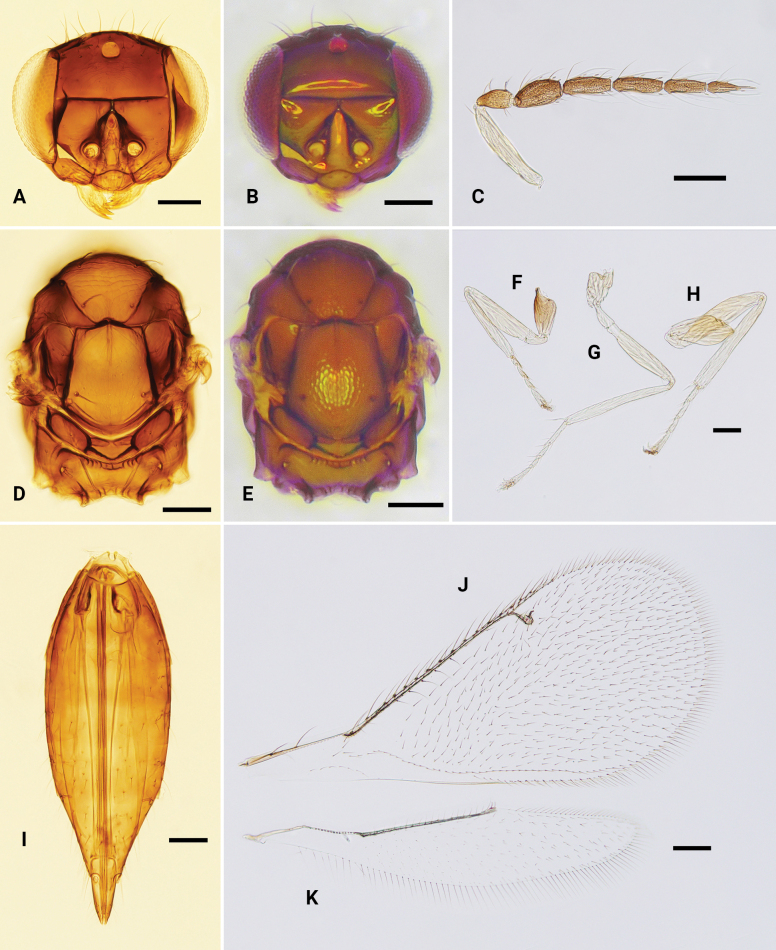
*Omphalerectisulcus* sp. nov., paratype, ♀ **A, B** head, frontal view **C** antenna **D, E** mesosoma, dorsal view **F–H** fore, mid and hind leg, respectively **I** metasoma, dorsal view **J** fore wing **K** hind wing. Scale bars: 100 μm.

***Head*** (Fig. 8A, B) in frontal view 1.2 × as wide as high; face and vertex smooth, only parts around antennal toruli with very weak reticulation and genae with weak transverse sculpture; occipital margin with a sharp edge; POL:OOL ~ 1.3:1.0; frontal sulcus straight, reaching eye margin, distance from frontal sulcus to median ocellus as long as distance to antennal toruli; antennal scrobes slightly wide and deep, meeting below frontal sulcus and connected to frontal sulcus by a short longitudinal suture; frontal cross-ridge slightly W-shaped and not reaching eye margin; clypeus with both upper and lower margins arcuate, nearly oval-shaped, 2.0 × as wide as high; mandible with two large teeth at apex and five smaller teeth at base; gena curved and slightly convex; HE:MS:WM ~ 5.7:1.0:4.7. Antenna (Fig. 8C) with all five flagellomeres separated from each other; scape 4.7 × as long as wide; pedicel 1.8 × as long as wide, and 0.75 × as long as F1; flagellomeres with F1, F2 and F3 almost equal in length, width of basal part of F1 equal to width of F2, but widest part of F1 1.5 × as wide as F2.

***Mesosoma*** (Fig. 8D, E) 1.5 × as long as wide; pronotum reduced and not visible in dorsal view; mesoscutum with shallow polygonal reticulation, meshes of reticulation barely elongate or transverse; notauli step-shaped, indicated by smooth depressions; median part of mesoscutum with two pairs of setae, and posterior margin slightly emarginate; mesoscutellum 1.2 × as long as wide, with same reticulation as mesoscutum, anterior 1/4–1/3 with a weak median groove, anterior corners impressed, single pair of setae located in the lower middle of scutellum; axillae with weak reticulation; metascutellum smooth, 0.4 × as long as wide, and 0.5–0.6 × as long as length of median propodeum, with two foveae anterolaterally; lateral panels of metanotum smooth; propodeum smooth with a narrow groove along anterior margin, without median carina. Fore wing (Fig. 8J) 2.3 × as long as wide, speculum closed; with seven admarginal setae arising from both MV and from membrane just below MV; PMV distinctly longer than STV, radial cell bare; ratio of SMV:MV:PMV:STV ~ 4.2:7.5:2.1:1.0. Hind wing (Fig. 8K) 4.8 × as long as wide, apex rounded. Legs (Fig. 8F–H) with metatibial spur nearly reaching apex of basal tarsomere.

***Metasoma*** (Fig. 8I) with petiole short and wide; gaster 1.9 × as long as length of mesosoma, and longer than head + mesosoma (1.2:1.0); ovipositor sheaths exserted beyond apex of gaster.

**Male.** Unknown.

##### Host.

Unknown.

##### Etymology.

The specific name refers to the straight frontal sulcus (*recti*- is Latin for straight).

##### Distribution.

China (Sichuan and Jiangxi Provinces).

##### Remarks.

*Omphalerectisulcus* sp. nov. should belong to the *huggerti* group, and is closest to *O.aperta* Hansson, 2004. The two species share the following characteristics: frontal sulcus straight or nearly straight; antennal scrobes meeting below frontal sulcus and connected to frontal sulcus by a short longitudinal suture; metascutellum flat, with two foveae anterolaterally; petiole short and wide. *Omphalerectisulcus* sp. nov. differs from *O.aperta* in having the clypeus nearly oval-shaped (vs nearly semicircular in *O.aperta*); fore wing with seven admarginal setae arising from MV and membrane just below MV (vs six in *O.aperta*), speculum closed (vs open below in *O.aperta*), PMV distinctly longer than STV (vs shorter than in *O.aperta*); propodeum without median carina (vs with median carina in *O.aperta*).

#### ﻿ Omphale sulciscuta

Taxon classificationAnimaliaHymenopteraEulophidae

(Thomson, 1878)

25DF9901-12F7-539F-9DFA-80285BA8D609

Figs 1I , 9A–K

Derostenus (Holcopelte) sulciscuta Thomson, 1878: 272, holotype ♀, Sweden, LUZN, not examined.
Horismenus sulciscuta (Thomson, 1878): Schmiedeknecht (1909): 433.
Holcopelte sulciscuta (Thomson, 1878): Bouček (1971): 541.
Omphale sulciscuta (Thomson, 1878): Hansson and Shevtsova (2012): 122.

##### Material examined.

• 2♀: 1♀ [NEFU; on card], China, Heilongjiang Province, Yichun City, Dailing District, Liangshui National Nature Reserve, 26.VII.2015, leg. Si-Zhu Liu, Xing-Yue Jin, and Xin-Yu Zhang, by sweep netting • 1♀ [NEFU; on slide], China, Heilongjiang Province, Yichun City, Dailing District, Liangshui National Nature Reserve, 02.VIII.2015, leg. Si-Zhu Liu, Xing-Yue Jin, and Xin-Yu Zhang, by sweep netting.

##### Diagnosis.

**Figure 9.**
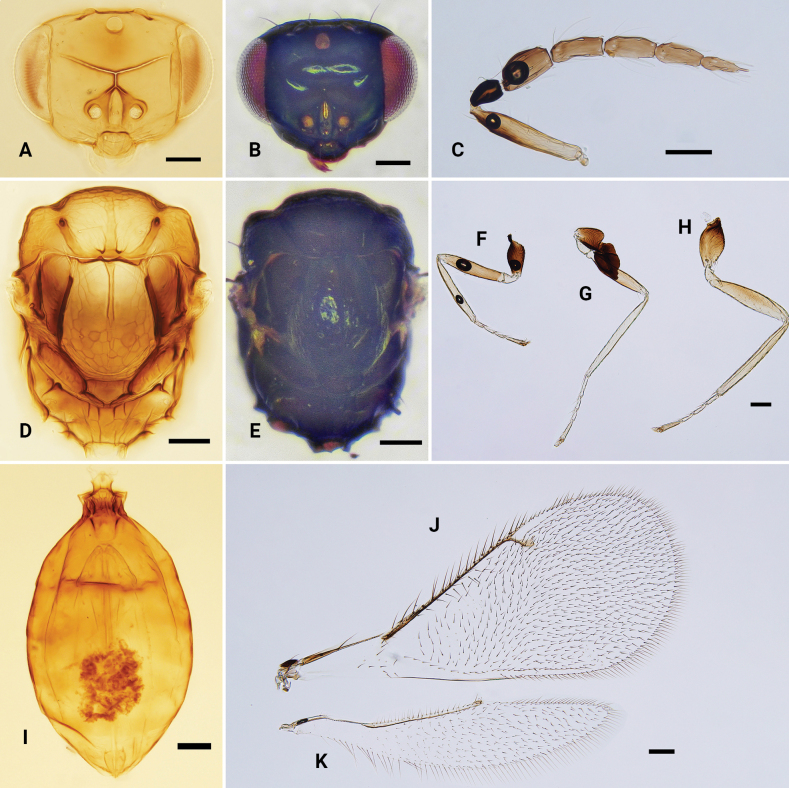
*Omphalesulciscuta*, ♀ **A, B** head, frontal view **C** antenna **D, E** mesosoma, dorsal view **F–H** fore, mid and hind leg, respectively **I** metasoma, dorsal view **J** fore wing **K** hind wing. Scale bars: 100 μm.

**Female.** Body length 1.1–1.7 mm, strongly sclerotized, mainly black to dark brown; antenna with scape yellowish brown to brown, pedicel and flagellum dark brown; fore wing hyaline. Head (Fig. 9A, B) with face and vertex smooth, frontal sulcus V-shaped, antennal scrobes meeting below meeting below frontal sulcus and connected frontal sulcus with longitudinal suture; clypeus trapezoid, 1.5 × as wide as high. Antenna (Fig. 9C) with five flagellomeres separated from each other; scape slightly stout, 5.2 × as long as wide; pedicel 1.5 × as long as wide; F1 1.0 × as long and 2.0 × as wide as F2; F1–5 with verticillate setae at base, the setae reaching or reaching beyond the apex of flagellomere attached to. Mesosoma (Fig. 9D, E) with mesoscutum and scutellum with distinct and sparse reticulation, meshes of reticulation mainly hexagonal; midlobe of mesoscutum with two pairs of setae and an incomplete shallow median groove; notauli as distinct deep grooves in posterior 1/2; scutellum 1.2 × as long as wide, with an incomplete median groove in anterior 1/2–2/3; metascutellum tongue like and without foveae anterolaterally; propodeum smooth, with a weak and narrow median carina, plica, between median carina and spiracular sulcus with a longitudinal carina, posterior part forming a short nucha; Fore wing (Fig. 9J) speculum closed, with 8–12 admarginal setae arising from MV and membrane just below MV; PMV shorter than STV, 0.7–0.8 × as long as STV, radial cell nearly bare (at least without setae along PMV). Metasoma (Fig. 9I), petiole quadratic to transverse, with anterior part drawn out to form a sharp margin that covers propodeal nucha; gaster 1.3 × as long as mesosoma, slightly longer than head + mesosoma (1.1:1.0).

**Male.** See Hansson and Shevtsova (2012).

##### Host.

Unknown.

##### Distribution.

China (Heilongjiang, Gansu, and Guangxi provinces); Armenia and Germany (Bouček and Askew 1968); Bulgaria (Boyadzhiev 2006); Croatia and Montenegro (Bouček 1977); Czech Republic (Bouček 1957); Denmark, France, and Russia (Hansson and Shevtsova 2012); Hungary (Erdös 1956); Moldova (Bouček 1965); Netherlands (Gijswijt 2003); Romania (Hansson 2016); Slovakia (Kalina 1989); Sweden (Thomson 1878); United Kingdom (Graham 1959).

#### ﻿ Omphale xanthosoma

Taxon classificationAnimaliaHymenopteraEulophidae

Li & Li sp. nov.

FE11B481-821F-5830-8BC1-816FF5B5E614

https://zoobank.org/B9396418-A6D1-4B45-BA54-AF51AF74BD45

Fig. 10A–F

##### Material examined.

***Holotype***: • ♀ [NEFU; on card, right antenna and right wings on slide], China, Hainan Province, Ledong Li Autonomous County, Jianfengling National Forest Park, 18.V.2021, leg. Ming-Rui Li and Gang Fu, by sweep netting.

##### Diagnosis.

**Female.** Body mainly yellow without metallic reflections, mesoscutum and mesoscutellum with a brown median stripe, posterior margin of abdominal tergites and apical part of ovipositor sheaths brown to dark brown; mid lobe of mesoscutum with only one pair of setae; gaster lanceolate, 2.0 × as long as length of mesosoma, obviously longer than head + mesosoma (1.6:1.0); antenna with scape short, 4.0 × as long as wide; fore wing with ten admarginal setae, radial cell nearly bare, with a sparsely hairline from the middle part of radial cell.

##### Description.

**Female.** Body length 1.4 mm, mainly yellow without metallic reflections, with a brown longitudinal stripe along median part of mesoscutum and mesoscutellum, posterior margin of abdominal tergites and apical part of ovipositor sheaths dark brown; eyes dull red; antenna with scape yellowish white, except apical 1/3 of dorsal edge brown, pedicel pale brown to brown, flagellum dark brown; mandibles with teeth dark brown; all legs yellow to yellowish white, except brown tarsal claws; wings hyaline.

**Figure 10.**
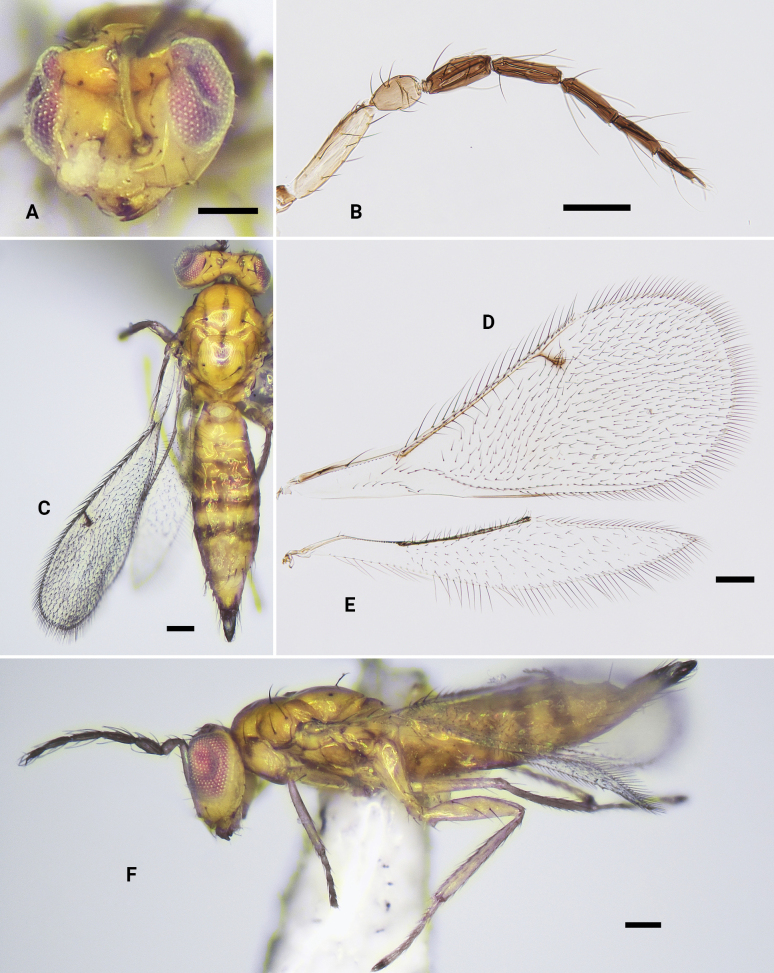
*Omphalexanthosoma* sp. nov., holotype, ♀ **A** head, frontal view **B** antenna **C** dorsal habitus, shows mesosoma and metasoma **D** fore wing **E** hind wing **F** lateral habitus, shows legs. Scale bars: 100 μm.

***Head*** (Fig. 10A) in frontal view 1.3 × as wide as high, slightly collapsed after death; face and vertex smooth; POL:OOL ~ 1.5:1.0; frontal sulcus arcuate, reaching eye margin, the midpoint closer to median ocellus than to antennal toruli; antennal scrobes join frontal sulcus separately; antennal toruli situated above level of lower eye margin; HE:MS:WM ~ 3.1:1.0:3.0; clypeus more or less semicircular, with upper margin weakly delimited and lower margin protruding, 2.2 × as wide as high; mandible with two large and pointed teeth at apex and one smaller and obtuse tooth at base. Antenna (Fig. 10B) with all five flagellomeres separated from each other; scape 4.0 × as long as wide; pedicel 1.4 × as long as wide; F1 1.0 × as long and 1.4 × as wide as F2; F3 to F5 slightly decreasing in both length and width.

***Mesosoma*** (Fig. 10C) 1.4 × as long as wide; pronotum reduced and not visible in dorsal view; mesoscutum with fine reticulation, mid lobe with only one pair of black setae; notauli indicated in anterior third; mesoscutellum 1.1 × as long as wide, anterior 1/2 with fine reticulation, posterior 1/2 smooth, with one pair of black setae located in the middle part; metascutellum small and triangular; propodeum short medially, 0.2 × as long as mesoscutellum, smooth, without median carina or plica. Fore wing (Fig. 10D) 2.5 × as long as wide, speculum closed, with ten admarginal setae arising from both MV and from membrane just below MV, with apical setae attached close to STV; PMV longer than STV, radial cell nearly bare, with a sparsely hairline from the middle part of radial cell, ratio of SMV:MV:PMV:STV ~ 3.9:5.3:1.9:1.0. Hind wing (Fig. 10E) 5.3 × as long as wide, apex pointed. Legs (Fig. 10F)with hind femur with three distinct long setae on outer side.

***Metasoma*** (Fig. 10C) 3.4 × as long as wide; petiole short; Gaster lanceolate, 2.0 × as long as length of mesosoma, obviously longer than head + mesosoma (1.6:1.0); ovipositor sheaths exserted beyond apex of gaster.

**Male.** Unknown.

##### Host.

Unknown.

##### Etymology.

The specific name refers to the yellow body of this new species.

##### Distribution.

China (Hainan Province).

##### Remarks.

*Omphalexanthosoma* sp. nov. should belong to the *obscurinotata* group, and is similar to *Omphalemellea* Hansson. The two species share the following characteristics: body mostly yellow; antenna with scape mainly yellowish white to yellow, flagellum dark brown; mid lobe of mesoscutum with only one pair of setae; metascutellum small; propodeum short medially; fore wing with radial cell nearly bare, with a sparsely hairline from the middle part of radial cell; gaster elongate. *Omphalexanthosoma* sp. nov. differs from *O.mellea* in having a brown longitudinal stripe along the median part of the mesoscutum and mesoscutellum (vs only scutellum occasionally with a median infuscate stripe in *O.mellea*); fore wing hyaline, without any infuscate part (hyaline, infuscate close to STV in *O.mellea*); antenna with flagellum slender, F2 and F3 both nearly as long as F1 (vs flagellum stouter, F2 and F3 both shorter than F1 in *O.mellea*). *Omphalexanthosoma* sp. nov. also looks similar to *O.melina* but can be easily separated from it through the one pair setae on the midlobe of the mesoscutum and the narrow STV (midlobe of mesoscutum with two pairs setae and STV enlarged in *O.melina*). *Omphalexanthosoma* sp. nov.is also similar to *O.ochra* Hansson & Shevtsova, 2012 and *O.rodopiensis* Yefremova, Yegorenkova & Boyadzhiev, 2017. Habitually, it can be easily separated from the latter two species through the mostly yellow and non-metallic mesoscutum and the long PMV, which 1.9 × as long as STV (mesoscutum with at least anterior 1/2 golden green, PMV 0.7–0.9 × as long as STV in both *O.ochra* and *O.rodopiensis*, see Yefremova et al. (2017)).

## Supplementary Material

XML Treatment for Omphale brevibuccata

XML Treatment for Omphale connectens

XML Treatment for Omphale longigena

XML Treatment for Omphale longitarsus

XML Treatment for Omphale melina

XML Treatment for Omphale obscura

XML Treatment for Omphale rectisulcus

XML Treatment for Omphale sulciscuta

XML Treatment for Omphale xanthosoma

## ﻿Additional information

### Author ORCIDs

Ming-Rui Li https://orcid.org/0000-0002-9143-1548

Qing-Fan Meng https://orcid.org/0009-0007-9729-7435

Sheng-Dong Liu https://orcid.org/0000-0002-4699-2469

### Data availability

All of the data that support the findings of this study are available in the main text.
